# Patient care activities by community pharmacists in a capitation funding model mental health and addictions program

**DOI:** 10.1186/s12888-018-1746-3

**Published:** 2018-06-14

**Authors:** Andrea L. Murphy, David M. Gardner, Lisa M. Jacobs

**Affiliations:** 1 College of Pharmacy and Department of Psychiatry, Dalhousie University, 5968 College St, PO Box 15000, Halifax, NS B3H 4R2 Canada; 2 Department of Psychiatry and College of Pharmacy, Dalhousie University, QEII HSC, AJLB 7517, 5909 Veterans’ Memorial Lane, Halifax, NS B3H 2E2 Canada; 3 Independent Evaluator, Contact Consulting, Halifax, NS Canada

**Keywords:** Mental disorders, Community pharmacy services, Pharmacists, Observational study

## Abstract

**Background:**

Community pharmacists are autonomous, regulated health care professionals located in urban and rural communities in Canada. The accessibility, knowledge, and skills of community pharmacists can be leveraged to increase mental illness and addictions care in communities.

**Methods:**

The Bloom Program was designed, developed, and implemented based on the Behaviour Change Wheel and a program of research in community pharmacy mental healthcare capacity building. We evaluated the Bloom Program as a demonstration project using mixed methods. A retrospective chart audit was conducted to examine outcomes and these are reported in this paper.

**Results:**

We collected 201 patient charts from 23 pharmacies in Nova Scotia with 182 patients having at least one or more follow-up visits. Anxiety (*n* = 126, 69%), depression (*n* = 112, 62%), and sleep disorders (*n* = 64, 35%) were the most frequent mental health problems. Comorbid physical health problems were documented in 57% (*n* = 104). The average number of prescribed medications was 5.5 (range 0 to 24). Sixty seven percent (*n* = 122) were taking multiple psychotropics and 71% (*n* = 130) reported taking more than one medication for physical health problems. Treatment optimization was the leading reason for enrollment with more than 80% seeking improvements in symptom management and daily functioning. There were a total of 1233 patient-care meetings documented, of which the duration was recorded in 1098. The median time for enrolling, assessing, and providing follow-up care by pharmacists was 142 min (mean 176, SD 128) per patient. The median follow-up encounter duration was 15 min. A total of 146 patient care encounters were 60 min or longer, representing 13.3% of all timed encounters.

**Conclusions:**

Pharmacists work with patients with lived experience of mental illness and addictions to improve medication related outcomes including those related to treatment optimization, reducing polytherapy, and facilitating withdrawal from medications. Pharmacists can offer their services frequently and routinely without the need for an appointment while affording patient confidentiality and privacy. Important roles for pharmacists around the deprescribing of various medications (e.g., benzodiazepines) have previously been supported and should be optimized and more broadly implemented. Further research on the best mechanisms to incentivize pharmacists in mental illness and addiction’s care should be explored.

## Background

Pharmacists are ideally positioned in community pharmacies to improve patient outcomes in mental illness and addictions care. A recent International Pharmaceutical Federation (FIP) report provided a global overview of how pharmacists contribute to the care and well-being of people living with mental health and addictions problems, including through optimizing treatment outcomes, education, early detection, triage, collaboration, health promotion, policy development, and research [1]. Adherence support has often been identified as a key service pharmacists can offer to people with mental health and addictions problems, however the FIP report illustrates a much broader and more integrated role of the community pharmacist in mental health and addictions [1].

Pharmacists’ roles have become increasingly collaborative and clinically-oriented [2] in mental illness and addictions care. Roles have been evolving beyond that of the “drug expert” [3] with a modernized scope [4]. Previous systematic reviews document the improvements in patient satisfaction and outcomes for those with lived experience of illness, such as depression and psychosis, largely due to medication management activities of pharmacists [5, 6]. The evolution of pharmacists’ roles and enhanced clinical service delivery also provides the opportunity to narrow gaps in the mental health system including those related to effectiveness, efficiency, and equality and equity of care [7, 8]. These roles are possible and supported by a trusting public who frequently access pharmacists’ services [9–11]. However, impediments have been described that serve to undermine the potential value of community pharmacists in building on their contributions to mental health and addictions care in their communities [12–19].

In some regions, there is no expectation of complete privacy when visiting a pharmacy and pharmacist workflow demands in some settings can make it difficult to spend sufficient uninterrupted time with a patient [16, 20, 21]. Communication and location of healthcare providers offers an ongoing challenge, one familiar to community pharmacists [22]. Another important barrier, though often overlooked, is that of role expectation. People will have different expectations of their pharmacists, often based on past interactions [23–25]. Role modification can therefore be met with a range of responses [26] taking substantial time and effort to establish comfort and confidence.

Nova Scotia is a province in eastern Canada of approximately 949,500 people [27] served by 303 community pharmacies [28]. Many (urban = 99.2%, rural = 53.3%) Nova Scotians live within five kilometres of a community pharmacy [10], making pharmacists accessible healthcare providers, even in rural areas. In 2012, the provincial government released a mental health and addictions strategy [29]. The strategy included 61 recommendations, of which more than 20 were amenable to pharmacists’ involvement and interventions. These included areas such as health promotion and early intervention; knowledge, education, and awareness of the public; collaboration with primary care providers; and access to services [29].

Based on the accessibility of pharmacists with expanding scopes and capabilities [30, 31], a progressive movement supported by government and the public to improve mental illness and addictions services, and an expanding, contextually relevant evidence base in this area [16, 19–21, 32, 33], we designed, developed, and implemented the Bloom Program – the mental health and addictions community pharmacy partnership program of Nova Scotia [34]. The Bloom Program was implemented as a demonstration project with an evaluation to facilitate decision-making around the program's future. In this paper, we describe the program, care activities provided, and outcomes of the Bloom Program demonstration project. Our analyses and inferences are primarily based on our patient chart reviews that served as one component of a broader mixed methods evaluation of the Bloom Program.

## Methods

### Design

**Fig. 1:**
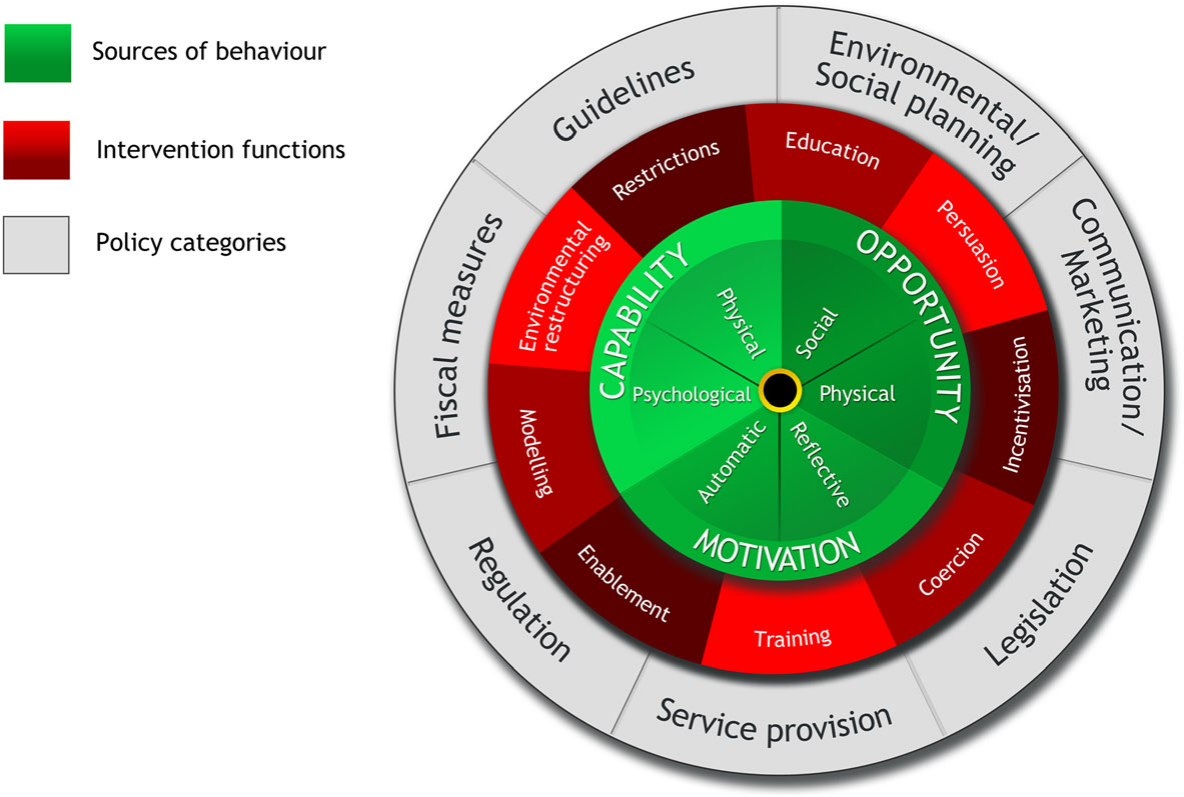
The Behaviour Change Wheel (BCW) [36]

**Fig. 2:**
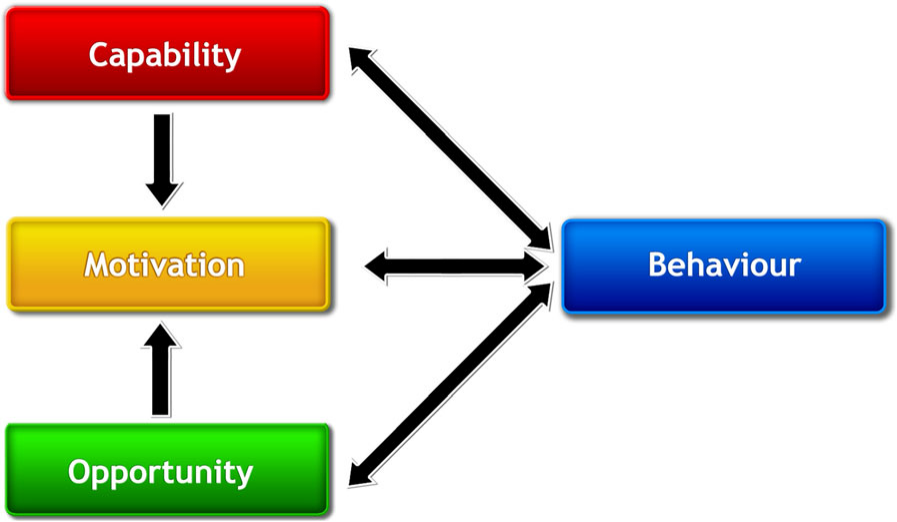
The Capability Opportunity Motivation – Behaviour (COM-B) model [36]

The Bloom Program design was underpinned by a theoretical model of behaviour, the Behaviour Change Wheel (BCW) (Fig. 1) [35, 36], which we used in our previous mental health program development [33]. The BCW was developed from 19 frameworks of behaviour change by Michie and colleagues [36]. At the centre of the BCW, there is a “behaviour system” that includes capability (C), opportunity (O), and motivation (M), which ultimately interact to produce behaviours (B) (COM-B) (Fig. 2). The BCW also includes nine intervention functions and seven policy categories that support intervention design (Fig. 1) [36].

The Theoretical Domains Framework (TDF) domains are mapped to the COM-B component of the BCW [35]. The TDF domains can be used to categorize or explain influences on behaviour and can be linked to the elements of COM-B to determine what intervention functions and/or policy categories may be best suited to the intervention design for changing target behaviours in context. Informed by the BCW, the core components of the Bloom Program, such as conducting community outreach and forming linkages with community mental health and addictions services and supports, and collaborating with other health care providers such as family physicians and psychiatrists, were intended to support pharmacists in their care of patients with lived experience of mental illness and addictions (Table 1).

**Table 1:** Bloom Program components

Component	Component description
1. Linkages	Developing and maintaining linkages with community mental health organizations.
2. Outreach	Providing outreach activities by the pharmacy and its pharmacists to support the local mental health community.
3. Collaboration	Enhancing collaboration and communication with other health providers, especially primary care and mental health and addictions care services.
4. Resources	Developing a local mental health knowledge exchange “resource centre”.
5. Training	Providing program-related education and training to all pharmacy team members.
6. Patient registration	Enrolment of targeted eligible patients by pharmacists with the program.
7. Enhanced patient care	Providing enhanced patient support services including: – Mental health and addictions systems navigation, resources and access support – Triage of care to appropriate health providers as indicated – In depth medication therapy management involving enhanced monitoring and overall assessment of addictions and mental illness as well as physical health disorders and their treatments – Collaboration with patients, families and other care providers to identify and resolve mental and physical health problems – Education consultations regarding mental health disorders and their treatment – Real-time support in person or via telephone during posted pharmacy operations
8. Quality assurance	Pharmacies participating in the program will maintain records demonstrating adherence to the program’s critical components. Participating pharmacies will apply to continue with the program every 2 years.
9. Program evaluation	A comprehensive evaluation of the Bloom Program.

Additionally, the program was supported by a capitation funding model in which pharmacies received a fixed  fee of $75/month Canadian (CAD) up to six times ($450/patient CAD) per patient. Fees were paid only during the months when care from pharmacists were received by the patient. If care was interrupted for longer than a month (e.g., due to hospitalization) no fee was paid to the pharmacy. The model of care was longitudinal with regular visits that addressed medications and related health issues (physical, mental, and addictions) that were collaboratively prioritized by the patient, pharmacist, and other members of their circle of care. If continued enrollment was indicated in the pharmacist’s judgment, in which new or ongoing medication issues existed, pharmacists could apply to extend a patient’s enrollment for another six month fee period at a lower compensation rate of $30/month CAD. Financial support to implement the Bloom Program and conduct an evaluation was provided by monies allocated through the provincial mental health and addictions strategy fund. The number of participating pharmacies and patients were limited based on the funding available.

Governance of the Bloom Program included a multistakeholder steering committee consisting of community members representing people with lived experience of mental illness, pharmacists, physicians, and representatives of the regional health authority, government, and professional advocacy and regulatory organizations of Nova Scotia. A Bloom Program evaluation subcommittee also provided feedback on the evaluation framework, data collection tools, and plans. The Program was implemented as a two-year demonstration project to ensure fitness for purpose.

### Bloom program demonstration project timeline

The demonstration project was completed over 27 months, starting September 2014 and ending for the evaluation of December 2016. The program continued without interruption following the demonstration project’s completion.

#### Pharmacy participants

**Fig. 3:**
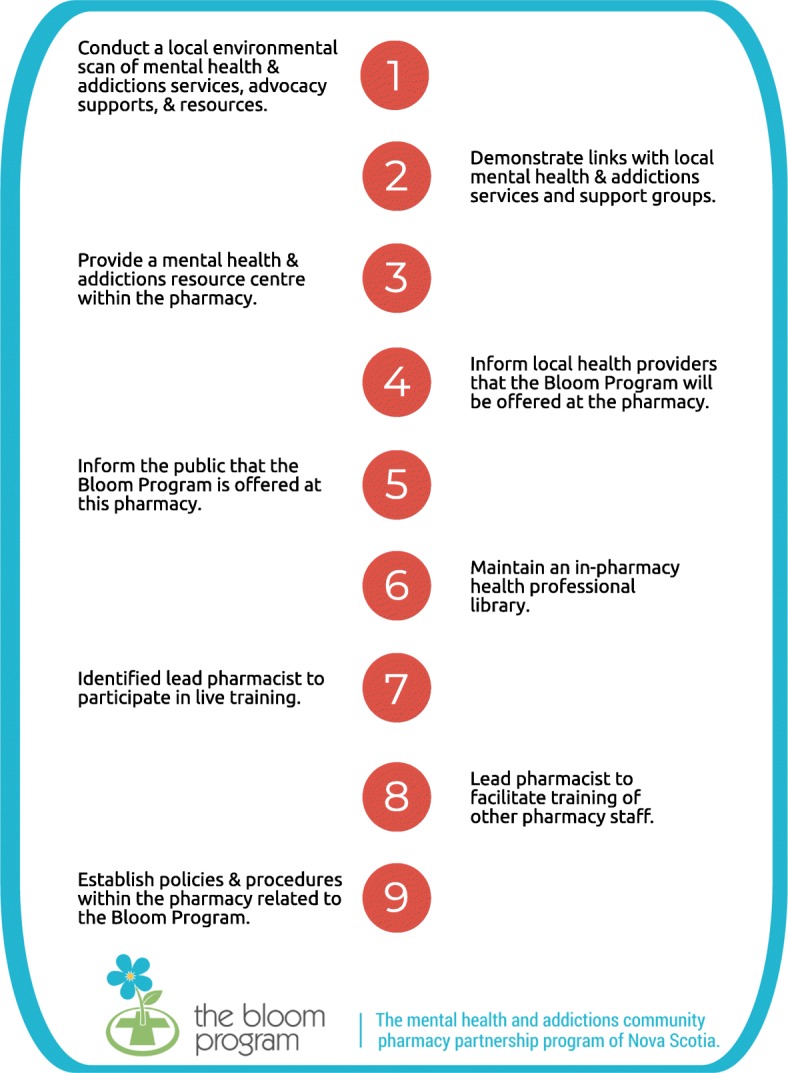
Nine-step Bloom Program pharmacy application

A designated lead pharmacist completed a 9-step application process (see Fig. 3) and received approval from the program administrator before the pharmacy was eligible to offer the Bloom Program. An additional criterion was the availability of a fully private consultation room in the pharmacy. To initiate the application process, lead pharmacists either expressed interest online or were informed of the opportunity based on their participation in previous community pharmacy mental illness focused initiatives [33, 37]. Twenty-three pharmacies were recruited to participate over 18 months via three training waves and were informed that a maximum of 20 patients per pharmacy could be enrolled at any one time.

#### Patient participants

There were two considerations to determine a patient’s eligibility: the presence of one or more self-identified mental illnesses and the existence of one or more medication-related issues. The list of eligible diagnoses was based on DSM-5 diagnostic categories and was intentionally broad and inclusive (Table 2). Pharmacists were to prioritize enrolling “high priority” patients, which were established based on illness prevalence, frequency of medication-related issues, knowledge of health service needs, and recognition of the important role pharmacists have in the care of people with these diagnoses. The aim was to enroll 70% or more people with one or more high priority diagnoses. Including the “other diagnoses” group ensured vertical equity of services for more vulnerable groups [38–40] who also have health and medication issues that are within the scope of a pharmacist’s practice.

**Table 2:** High priority and other diagnoses for Bloom Program enrollees

High priority diagnoses:	
Psychosis (e.g., schizophrenia, unspecified psychosis) Bipolar and related disorders (e.g., bipolar disorder types I and II) Depressive disorders (e.g., major depressive disorder) Anxiety disorders (e.g., social anxiety disorder, panic disorder) Obsessive-compulsive and related disorders (e.g., OCD, body dysmorphic disorder) Trauma and stress related disorders (e.g., post-traumatic stress disorder)	
Other diagnoses:	
Feeding and eating disorders (e.g., anorexia nervosa, bulimia nervosa) Sleep-wake disorders (e.g., insomnia disorder with episodic, persistent, or recurrent specifier (excluded is acute insomnia), narcolepsy, circadian rhythm sleep-wake disorders) Personality disorder (e.g., borderline personality disorder) Neurodevelopmental disorders (e.g., intellectual disability disorder, Autism, attention-deficit/hyperactivity disorder, tic disorder) Disruptive, impulse-control, and conduct disorders (e.g., oppositional defiant disorder, intermittent explosive disorder, conduct disorder) Substance-related and addictive disorders (e.g., alcohol use disorder; sedative, hypnotic, or anxiolytic use disorder)	

Pharmacists were to indicate which of the five medication-related issues existed at a patient’s enrollment (Table 3). Following enrollment, pharmacists and patients met and completed a comprehensive initial assessment and developed a plan to address a prioritized list of health and medication issues. Follow-up care was provided at scheduled meetings and/or convenience meetings (e.g., patient visit or telephone call to the pharmacy) and was determined based on a mutually agreeable schedule of the patient and pharmacist.

**Table 3:** Medication therapy issue eligibility criteria for Bloom Program patients

**Treatment optimization:** Following a standard trial of recent mental health/addictions pharmacotherapy, there is non-response or partial response requiring change in pharmacotherapy.	
**Treatment adverse effect:** Experiencing a treatment-limiting adverse effect to current mental health or addictions medication(s) requiring change in pharmacotherapy.	
**Non-adherence:** Medication refusal or non-adherence leading to a current or a near-recent decompensation of mental illness or addiction.	
**Medication withdrawal:** Difficulty tapering and stopping treatment for a mental health or addictions problem in a stable patient.	
**Inappropriate polytherapy:** Taking multiple medications, including psychotropics and non-psychotropics, that is causing functional impairment requiring modifications including medication discontinuation(s) on the basis of safety, redundancy, and absence of indication.	

#### Documentation, data collection, and analysis

The Bloom Program evaluation was conducted using mixed methods with various sources for data collection (e.g., surveys, interviews, enrolment forms, chart abstractions). This paper focuses on quantitative analyses for the retrospective chart review data that was collected. Other results and findings, such as those from qualitative analyses of interviews, will be reported elsewhere.

Participating pharmacies were provided with a complete set of forms to facilitate and document patient care and collaboration activities. Examples of these forms include: enrollment, initial assessment, progress notes, communications with healthcare providers, and discharge. Forms were constructed to support data collection and analyses. For example, check boxes were used to capture enrollment criteria and pharmacists’ actions related to follow-up visits. Activity duration was included on all forms to capture pharmacist’s time investment. Outcomes of health and medication issues were collected from the discharge form. At discharge, patients were asked to complete a form by documenting the health and medication issues that were identified at the initial assessment and throughout their participation in the Bloom Program. They scored the outcome of each problem as resolved, improved, unchanged, or worse. The pharmacist summarized the actions taken to address each identified problem.

Copies of Bloom Program patient charts maintained at each pharmacy were anonymized by pharmacy staff and securely forwarded to the evaluation team. Data were abstracted and analyzed using descriptive statistics with SPSS and Microsoft Office Excel.

## Results

### Patient characteristics

We collected 201 patient charts from 23 pharmacies in Nova Scotia with 182 patients having at least one or more follow-up visits after the initial assessment with the pharmacist (Table 4).

**Table 4:** Demographics of Bloom Program patients

	All patients (*n* = 201)		Patients with ≥1 follow-up visit (*n* = 182)	
	Mean	SD	Mean	SD
Age	48.1	15.7	47.9	16.1
	n	%	n	%
Sex				
Female	120	59.7	114	62.6
Male	81	40.3	68	37.4
Living situation				
Family/friends	131	65.2	118	64.8
Alone	47	23.4	44	24.2
Group home	7	3.5	7	3.8
Other	4	2.0	4	2.2
Unknown	12	6.0	9	4.9
Marital Status				
Married/common law	83	41.3	75	41.2
Single	75	37.3	70	38.5
Separated/ divorced	25	12.4	22	12.1
Unknown	18	9.0	15	8.2
Occupational status				
Employed	71	35.3	68	37.5
Unemployed	99	49.3	87	47.8
School	11	5.5	10	5.5
Unknown	20	10.0	17	9.3
Education				
Less than high school	27	13.4	24	13.2
High school	46	22.9	39	21.4
College/university	65	32.3	61	33.5
Unknown	63	31.3	58	31.9
Medication coverage				
Public insurance	96	47.8	87	47.8
Private insurance	78	38.8	70	38.5
Cash	20	10.0	18	9.9
Unknown	7	3.5	7	3.8
Physician care				
Family physician	188	93.5	173	95.1
Psychiatrist	66	32.8	63	34.6
None	9	4.5	6	3.3

### Diagnoses

Among 182 patients with one or more follow-up visits, anxiety (*n* = 126, 69%), depression (*n* = 112, 62%), and sleep disorders (*n* = 64, 35%) were the most frequent patient-identified mental health problems, followed by substance use disorders (*n* = 29, 16%), post-traumatic stress disorder (PTSD) (*n* = 27, 15%), and bipolar disorder (*n* = 20, 11%) (Table 5). Comorbid physical health problems were documented in 57% (*n* = 104) (Table 5).

**Table 5:** Health status at enrolment into the Bloom Program

	All patients (*n* = 201)		Patients with ≥1 follow-up visit (*n* = 182)	
	Mean	SD	Mean	SD
Number of stated health problems	2.7	1.4	2.7	1.4
	n	%	n	%
Participants with mental health and/or addictions problems	201	100	182	100
Psychotic disorder	13	6.5	11	6.0
Bipolar disorder	23	11.4	20	11.0
Depressive disorder	126	62.7	112	61.5
Anxiety disorder	139	69.2	126	69.2
Obsessive compulsive disorder	15	7.5	15	8.2
Post-traumatic stress disorder	29	14.4	27	14.8
Eating disorder	8	4.0	8	4.4
Insomnia or other sleep disorder	72	35.8	64	35.2
Personality disorder	11	5.5	11	6.0
ADHD	13	6.5	13	7.1
Disruptive behaviour disorder	6	3.0	6	3.3
Substance use disorder	32	15.9	29	15.9
Number of mental health and addictions problems	487		442	
Participants with physical health problems	113	56.2	104	57.1
Pain and neurological disorders	77	38.3	72	39.6
Cardiovascular disease	56	27.9	53	29.1
Gastrointestinal disorders	29	14.4	22	12.1
Endocrine disorders	27	13.4	25	13.7
Respiratory disorders	21	10.4	18	9.9
Other	47	23.4	44	24.2
Number of physical health problems	257		234	
Substance use				
Nicotine	78	38.8	66	36.3
Alcohol	75	37.3	68	37.4
Marijuana	36	17.9	30	16.5
Opioids	23	11.4	19	10.4

### Reason for enrollment

The leading reason patients enrolled in the Bloom Program was for treatment optimization. Over 80% indicated that they were seeking improvements in symptom management and daily functioning through working more closely with their pharmacist in the program (Table 6). Far fewer patients identified other eligibility criteria for enrolling. Approximately one-quarter identified managing adverse effects and 11% indicated non-adherence as important issues. Almost 20% chose the Bloom Program to help simplify their treatment regimen due to concerns of inappropriate medications or to access support in stopping selected medications, usually sedative-hypnotics.

**Table 6:** Medication issues and medication use at enrollment in the Bloom Program

	All patients (*n* = 201)		Patients with ≥1 follow-up visit (*n* = 182)	
	n	%	n	%
Medication issues:				
Treatment optimization	162	80.6	148	81.3
Adverse effects	49	24.4	44	24.2
Non-adherence	22	10.9	15	8.2
Medication withdrawal	27	13.4	23	12.6
Inappropriate polytherapy	12	6.0	9	4.9
Medications:				
Antidepressants	145	72.1	130	71.4
Benzodiazepines-Z drugs	107	53.2	98	53.8
Antipsychotics	58	28.9	50	27.5
Mood stabilizers	21	10.4	19	10.4
Psychostimulants	12	6.0	12	6.6
Other Psychotropics	13	6.5	12	6.6
Opioids	24	11.9	23	12.6
Opioid replacement therapy	15	7.5	14	7.7
Multiple psychotropic medications	136	67.7	122	67
No psychotropic medications	9	4.5	7	3.8
≥ 1 physical health medications	142	70.6	130	71.4
	Mean	SD	Mean	SD
Number of current medications	5.4	4.0	5.5	4.1
Range of current medications	0 to 24		0 to 24	

### Medication use

Among program patients who had one or more follow-up visits with the pharmacist, the average number of regularly used prescribed medications was 5.5 (range 0 to 24) (Table 6). Sixty seven percent (*n* = 122) were taking multiple psychotropics and 71% (*n* = 130) reported taking more than one medication for physical health problems. The most commonly used psychotropic medications were antidepressants, benzodiazepines and related sedatives, and antipsychotics. Use of nicotine, alcohol, and marijuana were also common (Table 5).

### Length, frequency, nature of visits, and disposition

**Fig. 4:**
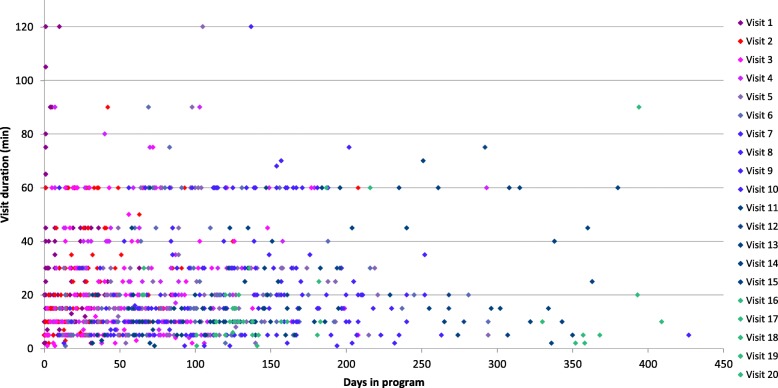
Density and duration of Bloom Program patient visits

The pattern of access varied for the 182 patients with one or more follow-up visit. There were a total of 1233 patient-care meetings documented of which the duration was recorded in 1098. Intensity of care was highest early in the program following patient enrollment with more frequent and longer visits that gradually declined as patients progressed through the program (Fig. 4). The median time invested by pharmacists in enrolling, assessing, and providing follow-up care (including direct patient care, communications with other patient care team members, and documentation) was 142 min (mean (SD) 176 (128)) per patient. The median follow-up encounter duration was 15 min.

There were a total of 146 patient care encounters of 60 min or longer, representing 13.3% of all timed encounters. Two pharmacies were outliers. They accounted for 101 (69%) of all longer patient encounters. In these two pharmacies, appointments of 60 min or longer accounted for 30 and 44% of patient encounters, respectively. In the other pharmacies, longer appointments of 60 min or more accounted for 4.5% of patient care encounters. Based on the visit pattern of the demonstration project, we estimated a reimbursement rate of $2.13 (CAD) per minute of the pharmacist’s time.

Patient disposition is shown in Table 7. Completed discharge forms were available for 46 participants. For this group, median duration in the program was six months (183 days, IQR: 155, 247). Thirty percent of patients were lost to follow-up during their participation in the program and 42% remained in the program. Seventeen requests for enrollment extensions were made by pharmacists with most relating to continued symptoms of mental illness (e.g., depression (*n* = 8), mixed symptoms of illnesses (*n* = 2), anxiety (n = 2), continued substance use (alcohol, tobacco) (*n* = 2), insomnia (*n* = 1)), and other reasons such as weight gain (*n* = 1) and discontinuation of medication (*n* = 1).

**Table 7:** Disposition of Bloom Program patients based on chart review^a^

Disposition	Number of patients (%)
Still in program	84 (41.8%)
Discharged using discharge form	46 (22.9%)
Assumed discharged (documented discharge plan with > 3 months of inactivity in Bloom Program)	11 (5.5%)
Early loss to follow-up (< 3 months in program)	37 (18.4%)
Late loss to follow-up (> 3 months in program without documented activity or planned discharge)	22 (11%)
Deceased^b^	1 (0.5%)
Total	201 (100%)

^a^Date of first patient enrolment: 20-Sep-2014. Date of last patient enrolment: 08-Mar-2016. ^b^ One frail elderly participant with multiple health issues died shortly after enrolling in the program

### Pharmacist patient care activities

**Fig. 5:**
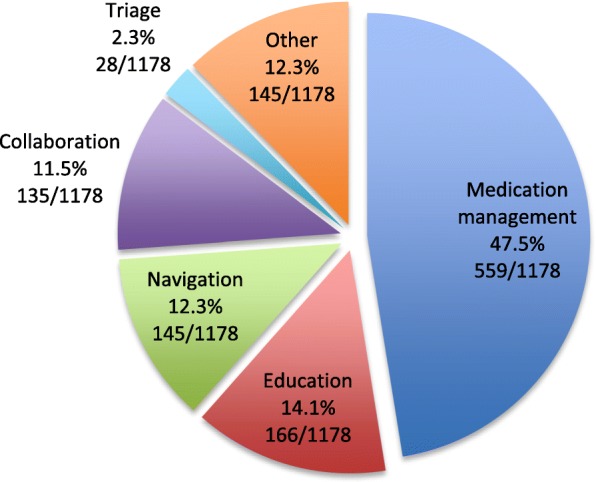
Purpose of follow-up visits between patients and pharmacists

The provided progress notes form, which was a semi-structured template for documentation of follow-up encounters, included check box options for pharmacists to indicate the actions taken related to the encounter. These included: medication management, navigation, education, collaboration, triage, and other. These forms were used in 1192 follow-up encounters. Documentation of encounters was evident for 90 other occasions in which the provided Bloom form was not used. A total of 1178 boxes were checked using the provided forms giving an estimate of the distribution of purposes and resulting actions of the follow-up meetings (Fig. 5). The most frequent activity and reason for follow-up meetings with pharmacists related to medication management. This accounted for nearly one half of the actions documented. Collectively, education, navigation, collaboration, triage, and other activities accounted for just over half of pharmacist patient care actions in the Bloom Program. To further elucidate the wider set of actions, we analyzed data from the first follow-up encounter for which 216 actions were recorded, 89 identified as medication management and 127 related to the other activities. There were 78 actions recorded when medication management was not identified as a purpose of the first meeting (navigation: 22, triage: 3, collaboration: 9, education: 21, and other: 23) indicating a high frequency of encounters between pharmacists and patients for purposes other than addressing medication issue. A more detailed analysis of these encounters is ongoing.

### Location of patient care activities and interactions

For patients with one or more follow-up visits, the pharmacist’s location was documented in 1039 encounters. The pharmacist was located at the pharmacy for 95.2% of the encounters and out of the pharmacy for 4.8% of encounters. This included encounters at physicians’ offices and the patients’ home. The telephone was used to facilitate 29.4% encounters, whereas the majority (70.6%) were in person. These data indicate that approximately two-thirds of encounters were face-to-face in the pharmacy’s private consultation room.

### Patient outcomes at Bloom Program discharge

**Fig. 6:**
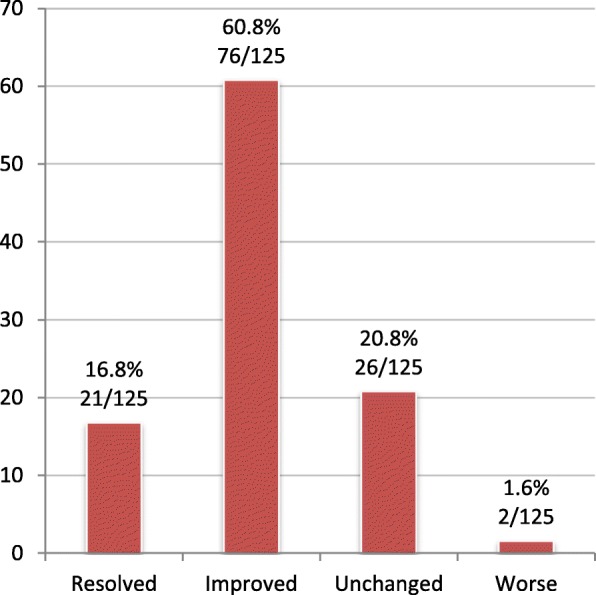
Patient-reported health problem outcomes at discharge (%)

Forty-six patients completed the Bloom Program discharge form with 125 health and medication problems identified and scored. The majority of problems (78%) were scored as “resolved” or “improved” at discharge from the Bloom Program (Fig. 6). Most patients in this subgroup entered the Bloom Program for treatment optimization (89%, 41/46).

Two of 125 problems were rated as “worse”. A patient with physical disability experienced weight gain in association with an increase in dose of olanzapine while participating in the program, rating the outcome of “weight loss” as worse. Another patient experienced a worsening of depressive symptoms in the first six months of the program and rated this problem as “worse”. During her enrollment, her pharmacist recommended starting an antidepressant to her family physician, the prescriber preferred to wait for mental health services involvement. In the interim, the patient experienced a personal crisis that led to a worsening of her mental health condition and a brief hospitalization. She was eventually started on antidepressant treatment with subsequent improvement and stabilization.

The health issues and actions taken to address the health issues as recorded by patients and pharmacists at discharge were varied and included activities other than those specific to medications (Table 8).

**Table 8:** Verbatim examples of discharge health and medication issue outcomes^a^

	Health issue	Action	Outcome
Treatment optimization	Anxiety & depression	Has improved through talking as well as having better control over asthma. Still feels defeated and drained with anxiety more at night. But overall she is better.	Improved
	Sleep difficulty	Melatonin and changed Effexor® [venlafaxine] schedule.	Improved
	Insomnia. Average sleep 3 h per night, multiple medications.	Sleep therapy (CBTi^b^), weaned off hypnotics.	Resolved
	Improve depression	Initiation of Cipralex®[escitalopram], monitoring for effectiveness	Improved
	Did not feel comfortable taking venlafaxine	Pharmacist contacted doctor to have patient switched to citalopram.	Resolved
	Depressive episodes related to menses	Increased Paxil® [paroxetine], augmented with Abilify® [aripiprazole], controlled menses via depo [medroxy]progesterone.	Improved
	Anxiety + OCD^c^ tendencies	CBT^d^ option identified and accessed	Improved
	Antidepressant ineffective	Sent letter to doctor. He did not act/respond on it.	Unchanged
	Pain control	Changed to long-acting Hydromorph Contin® [hydromorphone].	Improved
	Anxiety	Meditation, speaking with pharmacist during Bloom, speaking with doctor.	Improved
	Anxiety, anger, paranoia	No changes in medications. [Patient] feels like this program has helped a lot. She has decreased anxiety coming into pharmacy, talking to me about her health/personal and mental health issues and feels comfortable if she needs help in the future. Still experiencing anger and paranoia - Has talked to doctor about referral to psychiatrist.	Improved
	Chronic pain	Acupuncture, tried nortriptyline, massage, chiropractor, yoga	Unchanged
	Weight	Controlled asthma better, therefore allowing her to exercise more and discontinue prednisone.	Improved
	PTSD^e^	Sertraline 50 mg started	Unchanged
	Seasonal depression	Light therapy suggested to be continued	Improved
Adverse effects	Medication side effects	Met regularly to discuss medication side effects.	Improved
	Fatigue/insomnia	Still unable to work full days. Tamoxifen may be causal but continuing × 2 years.	Unchanged
	Sertraline side effect management	Zantac® [ranitidine] 150 mg once daily half hour before sertraline.	Resolved
	Decreased sex drive	Switched oral contraceptive.	Resolved
Non-adherence	Not testing [blood glucose] regularly because of finances	Now on 5 injections per day of insulin - > seeing clinic for supplies	Improved
	Not taking meds properly	More organized - and knows what they are for, but now ++^f^ financial issues	Unchanged
Medication withdrawal	Looking for a more natural approach/would like to stop all medications.	We discussed current medication but did not think it was a good idea to stop everything abruptly.	Unchanged
Inappropriate polypharmacy	Domperidone + Ezetrol^®^ [ezetimibe] not needed.	Contacted doc for discontinuation. [Patient] felt fine without those.	Resolved
	Reduction in pill load.	Change in meds.	Improved
	Unnecessary OTC^g^ products	Stopped	Resolved
Other	Finances, tax return.	Had an accountant go through papers and get things straightened out.	Resolved
	Had not seen doctor for a long time	Helped encourage visit to doctor’s office. Was able to get to doctor and to get blood work done.	Resolved
	Overlap in medications from 2 doctors	Both doctors made aware - > patient now keeping them both informed on what she’s on	Improved

^a^ Where medications were written verbatim as brand name or single source products, the generic name has been added in square brackets

^b^ CBTi: cognitive behavioural therapy for insomnia

^c^ OCD: obsessive compulsive disorder

^d^ CBT: cognitive behavioural therapy

^e^ PTSD: post traumatic stress disorder

^f^ ++: indicates increased or significant

^g^ OTC: over the counter

## Discussion

Our findings suggest that pharmacists worked closely and frequently with patients in a community pharmacy-based mental illness and addictions program to address mental and physical health issues jointly prioritized by patients and pharmacists. Medication optimization actions were at the core of the Bloom Program whereby patients directly engaged pharmacists to support changes in their medication regimen to improve symptomatic and functional outcomes. Pharmacists routinely engaged with patients for purposes that extended beyond medication management, including supporting health system navigation, health and medication education, collaboration with other health providers, and rapid triage of care to other health providers. It may be that some of these other activities did relate to medication management, for example education and collaboration, but pharmacists routinely advocated for and directly pursued their patients’ access to other services and supports via their navigational (e.g., referral to community groups and specialty clinics; locating and determining the cost for private psychotherapy) and triage activities. The application process that prioritized building stronger connections with local mental health and addictions services and support organization may have facilitated this. Pharmacists were provided with and oriented to existing and new mental health and addictions resources and directories and were required to identify and make direct connections with local providers and supports related to mental health and addictions. Some of these outreach and navigation roles that fall outside of activities explicitly linked to medication management services are often unrecognized but part of routine practice. More recently, these efforts have been acknowledged and explored through systematic reviews and other methods. Some specific areas of study have included general health coaching, walking groups and exercise programs, smoking cessation, weight management, emergency hormonal contraception, general health promotion or screening (e.g., alcohol problems, cholesterol), services for those who misuse or abuse drugs, and sexual health [3, 41–48]. This analysis of the Bloom Program helps to elucidate and quantify the breadth and extent of pharmacists’ activities that go beyond patient-centred medication management.

During the Bloom Program, patients and pharmacists met together privately, frequently, regularly, and for some patients these meetings were long. These frequent and sometimes lengthy visits contrast with usual care in which pharmacists’ encounters are usually brief and narrow in focus, for example during antidepressant initiation and prescription refills [13, 14, 49]. Carter et al. [23] showed that engagement with pharmacy services may depend on patient perceptions of how well pharmacists listened to them in previous encounters. Knox et al. [50] also found that patients desire efficient, consistent, and personalized pharmacy services. Patients who enrolled in the Bloom Program could build and strengthen relationships with community pharmacists to work towards common goals. This is supported by the frequency and length of patient encounters triangulated with patient feedback from surveys and interviews regarding the program (data available upon request) in which almost all respondents indicated that the quality of care was very good to excellent and that they would recommend the program to a friend. When satisfied with the quality of pharmacists’ care, people willingly build and sustain relationships with pharmacists [51]. The opportunity for private encounters, in person or by telephone, overcomes the privacy issues reported by others as a barrier to patient participation in community pharmacy delivered services [52].

In addition to treatment optimization, nearly one in five Bloom Program patients were seeking the pharmacists’ assistance in managing inappropriate polytherapy or medication withdrawal. Two-thirds of the patients were taking multiple psychotropic medications, along with 71% taking multiple physical health medications. The most commonly used psychotropic medications were antidepressants and sedative-hypnotics, including various benzodiazepines and zopiclone. The latter is particularly relevant given that incident and prevalent use of these medications in Canada continues to rise in adults and seniors and the well-established risks with short- and long-term use [53–55]. The Bloom Program therefore can be used to facilitate deprescribing of benzodiazepines and related medications that often require frequent brief encounters with patients, their pharmacists, and prescribers [56–58]. Pharmacists in the Bloom Program also engaged in resolving physical health and related medication problems for their patients. This is especially important in those with lived experience of mental illness and addictions given the established issues of poor accessibility to care, inequalities in health care service delivery, and relatively poorer physical health outcomes affecting this population [59–68].

Incentivisation was one of the behaviour change intervention functions that was used to support the implementation and sustainability of the Bloom Program as per the Behaviour Change Wheel and Theoretical Domains Framework [35, 36]. Lack of payment for services and problems with payment models have long been cited as barriers to service provision and uptake by pharmacists [69–72]. Data support that incentivized services can help to improve patient outcomes [73], although appropriate payment strategies and mechanisms in pharmacy practice remain largely heterogeneous with the optimal model unknown based on a recent systematic review [74]. Pharmacies were compensated $450 for patients who received pharmacist’s care over six months, consecutively or with interruptions if there were months in which the patient was unavailable to receive care. The compensation per minute for the pharmacists’ time in the Bloom Program, estimated at $2.13 (CDN), is in keeping with estimates of compensation for other fee for service clinical activities including medication reviews [75]. This may represent and underestimate as we were not able to estimate pharmacist’s time when providing standard care. The use of the capitation funding model in the Bloom Program was intended to support a more holistic and longitudinal approach to patient care in contrast to the typical fee-for-service structure in pharmacy practice. However, the capitation funding model requires further research given the age of some of the existing research in this area [76–79] and its co-existence with other payment models currently used in pharmacy practice. The extent to which the payment model influenced pharmacists in their recruitment and care of patients to the program is not known but should be explored given that other research of physicians using capitation and fee for service payment demonstrates selection of patients may occur based on their risk and complexity [80].

### Limitations

The Bloom Program was not designed as a controlled trial and therefore the results should be interpreted accordingly.

## Conclusions

Pharmacist work with patients with lived experience mental illness and addictions to improve medication related outcomes including those related to treatment optimization, reducing polytherapy, and facilitating withdrawal from medications. Pharmacists can offer their services frequently and routinely without the need for an appointment while affording patient confidentiality and privacy. Given the substantial use of psychotropic medications including polypharmacy, efforts should be made to leverage the strengths and skills of professionals such as pharmacists in efforts to facilitate medication management. Important roles for pharmacists around the deprescribing of inappropriate medications (e.g., benzodiazepines) have previously been supported and should be optimized and more broadly implemented. Further research on the best mechanisms to incentivize pharmacists in mental illness and addiction’s care should be explored.
